# A multi‐center, randomized, open‐label, parallel group study of a natural micronized progesterone vaginal tablet as a luteal support agent in Japanese women undergoing assisted reproductive technology

**DOI:** 10.1007/s12522-015-0211-y

**Published:** 2015-06-14

**Authors:** Toshihiro Fujiwara

**Affiliations:** ^1^ Center for Human Reproduction and Gynecologic Endoscopy, Sanno Hospital International University of Health and Welfare 8‐10‐16 Akasaka, Minato‐ku 107‐0052 Tokyo Japan

**Keywords:** Assisted reproductive technology (ART), FE 999913, Luteal support, Pregnancy rate, Vaginal insert

## Abstract

**Purpose:**

To verify sufficient progesterone supplementation during the luteal phase and to determine the efficacy and safety of a natural micronized progesterone vaginal tablet (FE 999913) in Japanese women undergoing in vitro fertilization‐embryo transfer or intracytoplasmic sperm injection.

**Methods:**

In a multi‐center, randomized, open‐label, parallel group trial, 108 subjects were randomized to receive FE 999913 vaginally twice daily (*n* = 54) or three times daily (*n* = 54) for up to 10 weeks. Primary endpoints were the proportion of subjects with serum progesterone concentration ≥10 ng/ml on day 5 and ongoing pregnancy rate at week 5.

**Results:**

Ninety‐four subjects completed the trial and 90 subjects underwent embryo transfer. The proportion of subjects with serum progesterone concentration ≥10 ng/ml on day 5 was 98.9 % and the lower limit of 95 % CI of the difference between the current trial and MEGASET trial was −3.6 %, which was within the non‐inferiority criterion. The ongoing pregnancy rate was 22.2 %, which is similar to that in actual clinical settings in Japan. No safety concerns were observed.

**Conclusions:**

FE 999913 was useful in this trial from the aspects of sufficient supplementation of progesterone, comparable pregnancy rate with that in clinical practice in Japan, and safety.

**Clinical trial registration:**

ClinicalTrials.gov identifier: NCT01710514.

## Introduction

Luteal support in assisted reproductive technology (ART) is important to maintain implantation after embryo transfer and to improve pregnancy rates. Luteal phase deficiency (luteal dysfunction) may result from controlled ovarian stimulation (COS) by hormonal management using gonadotropin‐releasing hormone (GnRH) agonists or GnRH antagonists in ART [1, 2, 3]. Therefore, hormone replacement with exogenous progesterone or activation of the corpus luteum by administration of human chorionic gonadotropin (hCG) is necessary for implantation after embryo transfer and maintenance of pregnancy. Luteal activation or luteal support with hCG improves pregnancy rates [4, 5], but may result in the onset of ovarian hyperstimulation syndrome (OHSS) [6, 7, 8, 9] and should therefore not be used by patients with a high risk of OHSS. Exogenous progesterone, on the other hand, does not cause OHSS [10]. Administration of exogenous progesterone is considered to be a useful method of luteal support therapy in ART treatment in Japan and worldwide.

Approved progesterone preparations in Japan are limited to injectable solutions and oral synthetic preparations indicated for the treatment of amenorrhea, dysmenorrhea, menometrorrhagia, infertility due to luteal phase defect, threatened abortion/premature labor, and habitual abortion. There are no approved vaginal progesterone preparations, nor approved progesterone preparations with the indication of luteal support in ART. Therefore, progesterone is used off‐label in Japan, mainly as progesterone intramuscular (IM) injections or oral synthetic progesterone preparations. Treatment with IM progesterone injections involves daily administration, which imposes a temporal and physical burden on patients. Daily injections are associated with a risk of injection site pain, nerve injury, and muscle contracture. The extent of the use of oral synthetic progesterone preparations in luteal support is unknown and for some of these preparations there are concerns regarding possible effects on the fetus [1]. Oral synthetic progesterone preparations are rarely used worldwide [11].

Foreign progesterone vaginal preparations are being unofficially imported or dispensed by the in‐house pharmacies at some medical institutions [12]. However, there are concerns regarding not only the quality of these preparations but also the development of adverse drug reactions and compensation and relief if such reactions develop. In the European Union and the US, vaginal progesterone preparations are used as a standard therapy for luteal support in infertility treatment [11, 12]. In Japan, however, there is an unmet medical need for progesterone supplementation during ART. Therefore, the Japan Society of Fertilization and Implantation and the non‐profit organization Fertility Information Network (Fine) filed a request for “addition of a vaginal dosage form for luteal supplementation during in vitro fertilization and embryo transfer (IVF‐ET)” to the Ministry of Health, Labor, and Welfare, which subsequently recruited a corporation to develop the requested vaginal preparation.

FE 999913 is a natural micronized progesterone effervescent vaginal tablet that contains 100 mg of progesterone per tablet as its active ingredient. It can be reliably administered by insertion vaginally using a dedicated applicator. As of October 2014, the FE 999913 vaginal tablet has been approved in 37 countries and territories worldwide including Japan, the US, and the UK, and is indicated for luteal support as part of an ART treatment program for infertile women.

The present investigation is a phase III clinical trial conducted to confirm the efficacy and safety of FE 999913 in an actual clinical setting in Japan.

## Materials and methods

This trial was a multi‐center, randomized, open‐label, parallel group phase III trial performed in 108 Japanese women of reproductive age undergoing IVF‐ET or intracytoplasmic sperm injection (ICSI).

The trial was designed to verify sufficient supplementation of progesterone during vaginal administration of 100 mg FE 999913 (LUTINUS; Ferring Pharmaceuticals) twice daily (BID) or three times daily (TID) and to determine the efficacy and safety of FE 999913.

The trial was conducted at six sites in Japan from October 2012 to August 2013 and was conducted in accordance with the Declaration of Helsinki and its amendments in force at the initiation of the trial, in compliance with the approved protocol, international conference on harmonization—good clinical practice, Japanese good clinical practice and applicable regulatory requirements. The trial was approved by an institutional review board at each site before the initiation of the trial.

### Trial population

Enrolled trial subjects were Japanese women of reproductive age who met the following inclusion criteria; (1) early follicular phase (day 2–4) follicle stimulating hormone concentration ≤12 IU/l and estradiol concentration <100 pg/ml; (2) luteinizing hormone, prolactin, and thyroid‐stimulating hormone within the normal limits for the clinical laboratory, or considered not clinically significant by the investigator within 6 months prior to screening; (3) a documented history of infertility [e.g., unable to conceive for at least 1 year (or for 6 months for women ≥38 years of age) or bilateral tubal occlusion or absence]; (4) transvaginal ultrasound (TVU) at screening (or within 14 days prior to screening) consistent with findings adequate for ART with respect to uterus and adnexa (peripheral reproductive organs); (5) at least one cycle with no fertility medication prior to screening; (6) hysterosalpingography, hysteroscopy, sonohysterogram, or TVU documenting a normal uterine cavity; (7) consent to contraception during the cycle in which pituitary down regulation was performed (prior to start of COS); and (8) signed informed consent to fertility treatment using FE 999913 after the subject and her husband had thoroughly understood the content.

Main exclusion criteria were the following: donor oocyte or embryo recipients; women with a body mass index (BMI) of >34 kg/m^2^; women with a poor response to gonadotropins; women with current or recent (within the past 12 months) substance abuse; women currently breast feeding, pregnant, or with a contraindication to pregnancy; women who refused to or were unable to comply with the trial procedures/conditions in the protocol; women who had participated in any experimental drug trial within 60 days prior to screening; women with a history of ≥3 spontaneous miscarriages and women with severe hepatic dysfunction or hepatic disease.

### Treatment regimen

COS was performed using the long GnRH agonist protocol or GnRH antagonist protocol according to the procedures of the trial site. Subjects meeting all eligibility criteria at screening were randomized to either the BID group or the TID group on the day of oocyte retrieval and administration of FE 999913 was initiated on the day following oocyte retrieval. Embryo transfer was performed on day 3 or day 5 after oocyte retrieval in accordance with the standard procedures of the trial site. Treatment with FE 999913 continued for up to 10 weeks if pregnancy was confirmed. A serum β‐hCG pregnancy test was performed at week 2 of treatment, and subjects were assessed for clinical pregnancy at week 4 of treatment and for ongoing pregnancy at week 5 of treatment.

### Trial endpoints

The primary endpoints were: (1) the proportion of subjects with serum progesterone concentration ≥10 ng/ml on day 5 of treatment and (2) the ongoing pregnancy rate (confirmation of fetal heart movements on TVU at week 5 of treatment). Only a subset of the randomized subjects had embryos available for transfer. The availability of embryos for transfer was not related to FE 999913. Therefore the evaluation of the ongoing pregnancy rate was based on the subjects who underwent embryo transfer. Secondary endpoints included the positive β‐hCG rate at week 2 of treatment, the clinical pregnancy rate (defined as presence of a gestational sac on TVU at week 4 of treatment) and the serum progesterone concentration throughout the treatment.

The safety of FE 999913 was evaluated by frequency and severity of adverse events, clinical laboratory tests (biochemistry, hematology, and urinalysis), 12‐lead electrocardiogram, physical examinations and gynecological examinations.

### Sample size and statistical analysis

This trial was powered with respect to the first co‐primary endpoint, the proportion of subjects with serum progesterone concentration ≥10 ng/ml on day 5 of treatment. The target was to demonstrate non‐inferiority against a historical control (the overseas MEGASET trial [13]), in which 631/632 (99.8 %) women had a progesterone concentration ≥10 ng/ml on day 5 of progesterone administration. The non‐inferiority limit was set to −10.0 % (absolute). Assuming that the probability of having a progesterone concentration ≥10 ng/ml is 98.5 % and using a non‐inferiority margin of −10.0 % (absolute), a total sample size of 80 subjects yielded more than 80 % power for establishing non‐inferiority using a two‐sided 95 % confidence interval (CI, corresponding to using a one‐sided alpha of 2.5 %).

The efficacy was analyzed using the full analysis set (FAS). For quantitative variables, the number of subjects, mean ± standard deviation (SD), and maximum and minimum are shown. For qualitative variables, the number of subjects and proportion of subjects in each category are shown. The first co‐primary endpoint was verified by comparing the proportion of subjects with serum progesterone concentration ≥10 ng/ml on day 5 of treatment observed in this trial to the proportion observed in the MEGASET trial [13] using a non‐inferiority argument based on the two‐sided 95 % CI. For the second co‐primary endpoint, the ongoing pregnancy rates at week 5 of treatment in the subjects with embryo transfer were calculated for each treatment group, along with two‐sided 95 % CIs. The positive β‐hCG rate and clinical pregnancy rate were analyzed in the same manner as the ongoing pregnancy rate. The intention‐to‐treat (ITT) population was included in the safety analysis. Safety parameters are summarized using descriptive statistics.

## Results

### Subject disposition

A total of 145 subjects were screened to participate in the trial, 37 subjects of whom were screening failures. The most common reason for screening failure was that inclusion/exclusion criteria were not met. Thus, 108 eligible subjects were randomized to receive FE 999913.

A total of 94 subjects (87.0 %) completed the trial and 14 subjects (13.0 %) discontinued the trial. The most common reason for discontinuation was an adverse event, with six subjects (11.1 %) in the BID group and seven subjects (13.0 %) in the TID group. One subject in the BID group discontinued due to withdrawn consent.

The ITT population included all 108 randomized subjects (54 in the BID group and 54 in the TID group). The FAS, defined as all randomized and exposed subjects with at least one completed primary efficacy assessment after treatment initiation, included 94 subjects (46 in the BID group and 48 in the TID group). The FAS with embryo transfer population included 90 subjects (43 in the BID group and 47 in the TID group).

### Baseline characteristics

In the FAS with embryo transfer population, subjects were Japanese women with a mean age of 34.7 years (range, 26–42 years), and the mean BMI was 21.3 kg/m^2^ (range, 16.0–28.0 kg/m^2^). The mean duration of infertility was 42.8 months (range, 9–137 months), and the most common cause of infertility was male factor (32 subjects, 35.6 %). The mean number of embryos transferred was 1.1. A total of 81 subjects (90.0 %) received one embryo and 83 subjects (92.2 %) underwent embryo transfer on day 3 after oocyte retrieval. Forty subjects underwent IVF‐ET and 50 subjects underwent ICSI. No differences were seen between treatment groups in terms of baseline characteristics (Table 1).

**Table 1:** Baseline characteristics—FAS with embryo transfer

Demographic data	FE 999913 BID	FE 999913 TID	Total
FAS with embryo transfer (*N*)	43	47	90
Age (years)			
Mean ± SD	35 ± 3.5	34.5 ± 4.3	34.7 ± 3.9
Range	26–42	26–41	26–42
Age group *N* (%)			
<35 years	16 (37.2 %)	21 (44.7 %)	37 (41.1 %)
35–37 years	16 (37.2 %)	14 (29.8 %)	30 (33.3 %)
38–40 years	10 (23.3 %)	7 (14.9 %)	17 (18.9 %)
41–42 years	1 (2.3 %)	5 (10.6 %)	6 (6.7 %)
BMI (kg/m^2^)			
Mean ± SD	21.2 ± 2.8	21.3 ± 2.9	21.3 ± 2.9
Range	16–27.9	17.2–28	16–28
Duration of infertility (months)			
Mean ± SD	42.1 ± 27.4	43.3 ± 23.1	42.8 ± 25.1
Range	9–137	13–101	9–137
Primary cause for infertility *N* (%)			
Unexplained	11 (25.6 %)	12 (25.5 %)	23 (25.6 %)
Tubal factor	16 (37.2 %)	14 (29.8 %)	30 (33.3 %)
Male factor (mild)	2 (4.7 %)	6 (12.8 %)	8 (8.9 %)
Male factor (moderate)	8 (18.6 %)	5 (10.6 %)	13 (14.4 %)
Male factor (severe)	2 (4.7 %)	9 (19.1 %)	11 (12.2 %)
WHO Group I anovulatory infertility	2 (4.7 %)		2 (2.2 %)
WHO Group II anovulatory infertility	1 (2.3 %)		1 (1.1 %)
Other	1 (2.3 %)	1 (2.1 %)	2 (2.2 %)
Day of transfer *N* (%)			
Day 3 embryo transfer	38 (88.4 %)	45 (95.7 %)	83 (92.2 %)
Day 5 embryo transfer	5 (11.6 %)	2 (4.3 %)	7 (7.8 %)
Subjects with embryo transferred *N* (%)			
1 embryo	39 (90.7 %)	42 (89.4 %)	81 (90.0 %)
2 embryos	4 (9.3 %)	5 (10.6 %)	9 (10.0 %)
Embryos transferred (mean ± SD)	1.09 ± 0.294	1.11 ± 0.312	1.1 ± 0.302
Method of insemination *N* (%)			
IVF	19 (44.2 %)	21 (44.7 %)	40 (44.4 %)
ICSI	24 (55.8 %)	26 (55.3 %)	50 (55.6 %)

*FAS* full analysis set, *BID* bis in die/twice daily, *TID* ter in die/three times daily, *SD* standard deviation, *BMI* body mass index, *WHO* World Health Organization, *IVF* in vitro fertilization, *ICSI* intracytoplasmic sperm injection

In the FAS, the mean duration of treatment (mean ± SD) was 27 ± 21 days, with a range of 3–72 days.

### Serum progesterone concentration

The proportion of subjects in the FAS with a serum progesterone concentration ≥10 ng/ml on day 5 of treatment was 98.9 % (95 % CI 94.2; 100.0, 93/94 subjects) (Table 2). In the MEGASET trial [13] this proportion was 99.8 % (95 % CI 99.1; 100.0, 631/632 subjects). The lower limit of the 95 % CI of the difference between the proportion of subjects with serum progesterone concentration ≥10 ng/ml on day 5 of treatment in the current trial and the MEGASET trial was −3.6 %, which exceeded the non‐inferiority criterion −10.0 % and therefore this primary objective was met.

**Table 2:** Proportion of subjects with serum progesterone concentration ≥10 ng/ml on day 5 of treatment‐FAS

FE 999913 (BID/TID)^a^				MEGASET trial progesterone preparation^b^				Difference^c^	
*N* ^d^	*N* ^e^	Estimate (%)	95 % CI^f^	*N* ^d^	*N* ^e^	Estimate (%)	95 % CI^f^	Estimate (%)	95 % CI^g^
94	93	98.9	94.2; 100.0	632	631	99.8	99.1; 100.0	−0.9	−3.6;1.8

*FAS* full analysis set, *BID* bis in die/twice daily, *TID* ter in die/three times daily

^a^Data from the BID group and TID group were pooled

^b^Historical control

^c^Difference = the current trial − the MEGASET trial

^d^Total number of subjects

^e^Number of subjects with serum progesterone concentration ≥10 ng/ml on day 5 of treatment

^f^95 % CI was calculated based on the exact Clopper–Pearson method

^g^95 % CI was calculated based on asymptotic normal distribution with a continuity correction

**Figure 1:**
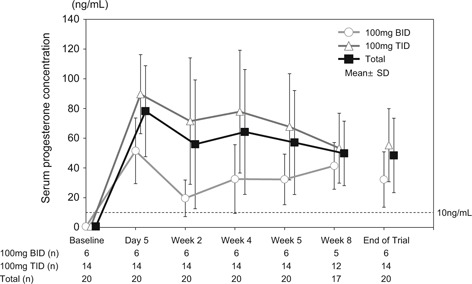
Serum progesterone concentration profile (mean ± SD) in subjects who were confirmed to be ongoing pregnant at week 5. This figure shows the mean serum progesterone concentration between day 5 and the end of trial in the 20 subjects in whom ongoing pregnancy was confirmed at week 5. In both treatment groups (100 mg BID: bis in die/twice daily, 100 mg TID: ter in die/three times daily), mean serum progesterone concentration sustained over 10 ng/ml throughout the treatment and no marked change was seen

The mean serum progesterone concentrations in the 20 subjects in whom ongoing pregnancy was confirmed at week 5 of treatment were 78.2 (day 5), 55.9 (week 2), 64.2 (week 4), 57.1 (week 5), 49.8 (week 8), and 48.4 ng/ml (end of trial). The mean serum progesterone concentrations sustained at the Japanese standard level during mid‐luteal phase (≥10 ng/ml) throughout the 10 weeks, and no marked changes were seen (Fig. 1).

### Pregnancy rates

In the FAS with embryo transfer population, the second co‐primary endpoint, the ongoing pregnancy rate at week 5 of treatment was 22.2 % (95 % CI 14.1; 32.2, 20/90 subjects) (Table 3). The clinical pregnancy rate was the same as the ongoing pregnancy rate; however, one pregnancy loss occurred in the BID group between week 2 and week 4.

**Table 3:** Ongoing pregnancy rates—FAS with embryo transfer

Treatment group	*N* ^a^	*N* ^b^	Estimate (%)	95 % CI^c^
Ongoing pregnancy rate				
BID	43	6	14.0	5.3; 27.9
TID	47	14	29.8	17.3; 44.9
Total	90	20	22.2	14.1; 32.2

*FAS* full analysis set, *BID* bis in die/twice daily, *TID* ter in die/three times daily

^a^Total number of subjects

^b^Number of subjects with confirmed ongoing pregnancy

^c^95 % CI was calculated based on the exact Clopper–Pearson method

Stratified analysis of the ongoing pregnancy rate in the FAS with embryo transfer was conducted using subgroups based on the day of embryo transfer, the number of embryos transferred, the primary cause for infertility, BMI, age, protocol type, and method of insemination.

There was no difference (*p* = 1.000, Fisher's exact test) in the ongoing pregnancy rate between the BID and TID groups in subjects younger than 35 years of age; 18.8 % (3/16 subjects) in the BID group and 14.3 % (3/21 subjects) in the TID group, while the ongoing pregnancy rate in the TID group was significantly higher in subjects 35 years or more; 11.1 % (3/27 subjects) in the BID group and 42.3 % (11/26 subjects) in the TID group (*p* = 0.014, Fisher's exact test) (Table 4).

**Table 4:** Ongoing pregnancy rates by age—FAS with embryo transfer

Treatment group	*N* ^a^	*N* ^b^	Estimate (%)	*p* value^c^
Ongoing pregnancy rate				
<35 years				
BID	16	3	18.8	1.000
TID	21	3	14.3	
≥35 years				
BID	27	3	11.1	0.014
TID	26	11	42.3	

*FAS* full analysis set, *BID* bis in die/twice daily, *TID* ter in die/three times daily

^a^Total number of subjects

^b^Number of subjects with confirmed ongoing pregnancy

^c^ *P* value was calculated based on Fisher's exact test

No distinct trends were observed in the ongoing pregnancy rates of the other subgroups.

### Safety

The overall incidence of adverse events was 35.2 % (38 subjects, 51 events) in the 108 subjects in the safety population. The incidence of serious adverse events (SAE) was 0.9 % (one subject, one event), and the incidence of adverse events that led to discontinuation was 8.3 % (nine subjects, nine events). The incidence of adverse events for which a causal relationship to FE 999913 could not be ruled out was 8.3 % (nine subjects, 12 events).

The most common adverse event in the safety population was OHSS at an incidence of 20.4 % (22 subjects). No cases of OHSS were judged to be severe in intensity, and the majority of cases were mild. A relationship to FE 999913 was ruled out for all cases of OHSS that occurred. Other common adverse events were abdominal distension (3.7 %, four subjects) and vaginal bleeding (3.7 %, four subjects).

One adverse event led to hospitalization (subchorionic hemorrhage), but was judged as not related to FE 999913 by the investigator.

The most common adverse events for which relationship to FE 999913 could not be ruled out were headache, somnolence, vaginal bleeding (1.9 %, two subjects each).

The profile of adverse events that were seen relatively frequently was similar between the BID group and the TID group.

Also, there were no clinically significant trends either within or between treatment groups with respect to clinical laboratory tests, vital signs, ECG, physical examination, and gynecological examination.

## Discussion

The current trial was the first randomized trial evaluating the efficacy and safety of vaginal administration of progesterone in Japanese women undergoing IVF‐ET or ICSI.

### Serum progesterone level with FE 999913

This trial demonstrated that the administration of FE 999913 100 mg BID or TID could maintain serum progesterone concentrations at ≥10 ng/ml, which is the lower reference level of the mid‐luteal phase criterion in Japanese women. FE 999913 also maintained a high serum progesterone concentration (mean values ~50 ng/ml) throughout the treatment period in subjects who became pregnant.

The proportion of subjects with a serum progesterone concentration of ≥10 ng/ml on day 5 of treatment was compared with the results from an overseas clinical trial (MEGASET trial [13]). The proportion of subjects with serum progesterone concentration ≥10 mg/ml on day 5 of treatment in the current trial fulfilled the non‐inferiority criterion relative to the results of the MEGASET trial. Furthermore, the daily dosage of progesterone used in the current trial was 200 or 300 mg. Nonetheless, non‐inferiority to the vaginal high‐dose (600 mg) progesterone capsule that was used in the MEGASET trial (UTROGESTAN: not approved in Japan) was verified.

### Pregnancy rate of FE 999913

The ongoing pregnancy rate per embryo transfer at week 5 of treatment in this trial was 22.2 % (20/90 subjects; BID group: 14.0 %, TID group: 29.8 %). This pregnancy rate is comparable to the pregnancy rates in Japanese clinical trials of the already approved ART drugs FOLLISTIM injection and GANIREST subcutaneous injection (22.9 %; 35/153 subjects and 25.6 %; 20/78 subjects, respectively) [14, 15].

The clinical pregnancy rate at week 4 of treatment (22.2 %, 20/90 subjects) was comparable to the pregnancy rates listed in the “Report of the Registration and Survey Subcommittee of the 2012 Ethics Committee” of the Japan Society of Obstetrics and Gynecology (20.8 %; IVF‐ET: 22.6 %, ICSI—ejaculation sperm: 19.0 %, ICSI—testicular sperm extraction sperm: 15.3 %) [16].

Although the objective of this trial was not to compare and determine the efficacy of the two dose regimens, a numeric difference in the ongoing pregnancy rates was observed between the BID group (14.0 %, 6/43 subjects) and the TID group (29.8 %, 14/47 subjects). There were no differences in demographic variables between the two treatment groups. The ongoing pregnancy rate varied from 10 to 57 % depending on the site, and some sites had pregnant subjects only in the BID group or the TID group. Thus, it appears that there was no essential difference in the pregnancy rate between the BID and the TID groups, and the numeric difference between two groups was incidental. In addition, the ongoing pregnancy rate in subjects 35 years or more (42.3 %, 11/26 subjects) was higher than that in subjects younger than 35 years (14.3 %, 3/21 subjects) in the TID group. This numeric difference between two strata was also considered as incidental. Thus, the relatively small number of subjects in the current trial precludes drawing conclusions with regard to this observation. Further investigations are required to accumulate and evaluate more data in the future.

In a previous US multi‐center, randomized, open‐label, parallel group trial for the progesterone vaginal tablet (ENDOMETRIN: trade name of FE 999913 in the US), ENDOMETRIN was compared to a progesterone gel (CRINONE 8 % gel) in women undergoing IVF‐ET or ICSI [17]. This trial included 1211 patients and concluded that both ENDOMETRIN 100 mg BID and TID were non‐inferior to progesterone gel with respect to ongoing pregnancy rate. The ongoing pregnancy rates in this trial ranged from 38.6 to 42.3 % in the three treatment groups, which is in line with the US registry data [18]. The different ongoing pregnancy rates between the current Japanese trial and the US trial may partly be due to differences in baseline characteristics. The mean number of embryos transferred was 1.1 in the Japanese trial and 2.4 in the US trial. Only 8 % of the embryo transfers were performed on day 5 in the Japanese trial as compared to 30 % or more in the US trial.

### Safety of FE 999913

Adverse events occurred in 38 of 108 subjects (35.2 %). The most common adverse event was OHSS, which occurred in 22/108 subjects (20.4 %). However, OHSS is an adverse event associated with COS and is not related to FE 999913. The majority of adverse events were mild and transient and no clinically important events were noted.

In this trial, no new safety issues of concern were observed that would affect the known safety profile of FE 999913. No major safety‐related problems were identified, and FE 999913 was well tolerated.

### Usefulness of vaginal administration

Preparations of vaginal progesterone include gel, suppositories, and micronized progesterone vaginal inserts. However, as described previously, no progesterone preparations, including vaginal preparations, are approved for the indication of luteal support in ART in Japan. Therefore, injectable progesterone solutions, oral synthetic progesterone preparations, or progesterone vaginal preparations, unofficially imported or dispensed by in‐house pharmacies, are currently used. Treatment with progesterone injections is time‐consuming and requires daily visits to the hospital. Additionally, daily injections are associated with a risk of injection site pain, nerve injury, and muscle contracture. In the case of imported progesterone vaginal preparations, there are concerns regarding compensation and relief if adverse drug reactions should occur.

Vaginally administered FE 999913 overcomes the disadvantages of progesterone IM injections and will likely improve patient adherence. Additionally, the concerns regarding the use of imported vaginal preparations are addressed. Furthermore, while the vaginal administration of FE 999913 does not yield high serum concentrations of progesterone as seen with IM progesterone injections, higher concentrations can be achieved in the endometrium than with IM administration [19, 20, 21]. The mechanism responsible is considered to be the direct delivery of progesterone from the vagina to the uterus (uterine first‐pass effect) [22, 23]. As a result, a reduction in systemic adverse drug reactions associated with high serum concentrations was achieved [24, 25, 26].

Gel preparations accumulate a significant vaginal build‐up that can be uncomfortable for patients and may induce vaginal irritation [26]. Meanwhile, the dedicated applicator enables the delivery of FE 999913 into the depth of the vaginal cavity. This shortens the distance to the target organ (endometrium) and resolves the issues seen with the gel preparations such as discomfort with gel accumulation, vaginal irritancy, and the necessity for frequent hand washing.

FE 999913 has been useful overseas as a luteal support therapy in ART. Results of a nationwide survey of infertile patients in US undergoing ART, such as IVF, revealed that patients prefer ENDOMETRIN^®^ vaginal inserts to progesterone‐in‐oil injections, and to all other vaginal progesterone supplements. The reasons cited most frequently for their preference included easy, convenient, and pain‐free administration [27].

### Use of FE 999913 for frozen embryo transfer

In current clinical practice in Japan, frozen embryo transfer using hormone replacement is frequently performed. The 2012 Ethics Committee Registration/Surveillance Subcommittee Report (Clinical data for IVF/ET etc. in 2012 and registered institutions as of July 2014) reported that the efficacy of frozen embryo transfer is higher than that for fresh embryo transfer [16], with pregnancy rates of 33.7 % following frozen embryo transfer as compared to 20.8 % following fresh embryo transfer. In addition, according to the Japanese nationwide registry data from 2008 to 2010 [28], pregnancy rates after single embryo transfer are 19.0 % in fresh cleaved embryo transfer, 30.0 % in fresh blastocyst transfer, 19.4 % in thawed cleaved embryo transfer, and 38.5 % in thawed blastocyst transfer. FE 999913 is expected to be used in frozen embryo transfer cycle using hormone replacement, but further investigations are needed to confirm the efficacy and safety of FE 999913 in frozen embryo transfer cycles.

## Conclusions

The administration of FE 999913 BID or TID up to 10 weeks in Japanese women undergoing fertility treatment with IVF‐ET or ICSI could sufficiently supplement and maintain progesterone concentrations throughout the treatment period. Additionally, it was confirmed that the pregnancy rate was similar to that of already approved Japanese ART drug preparations, and similar to the pregnancy rate in actual clinical settings in Japan. The safety results from the current trial were consistent with the known safety profile of FE 999913 identified in previous overseas clinical trials. Thus, treatment with FE 999913 was safe and well tolerated in Japanese women undergoing IVF‐ET or ICSI.

Finally, there is a significant need for the use of a vaginal progesterone preparation with documented efficacy and safety in Japanese clinical practice. The use of FE 999913 in Japan is therefore expected to contribute greatly to fertility treatment in Japan and to allow luteal support with a safe, efficacious, and convenient therapy. Indeed, once FE 999913 is launched with an indication of “luteal support in ART” in Japan, it is expected that FE 999913 will become the most useful agent for luteal support in ART.

## Acknowledgments

The author wishes to thank all the principal investigators (PI), sub‐investigators, and other staff at the participating centers in Japan: (1) Sanno Hospital, Tokyo (PI: Takako Kurosawa); (2) Bashamichi Ladies’ Clinic, Yokohama (PI: Hideyuki Ikenaga); (3) Sophia Ladies’ Clinic, Sagamihara (PI: Yoshiaki Sato); (4) Hanabusa Women's Clinic, Kobe (PI: Masahide Shiotani); (5) IVF Namba Clinic, Osaka (PI: Keijiro Ito); and (6) Ebina Ladies’ Clinic, Ebina (PI: Yoshihito Kondo). The author would also like to thank Professor Osamu Ishihara, MD, PhD, Saitama Medical University for trial coordination. This clinical trial was sponsored by Ferring Pharmaceuticals Co., Ltd.

### Conflict of interest

The author acts as a medical advisor of this clinical trial and consultant for Ferring Pharmaceuticals. The author also acts as a consultant for ASKA Pharmaceutical. The author receives an unrestricted educational grant from Merck Serono and Mochida Pharmaceutical.

### Human rights statements and informed consent

All procedures followed were in accordance with the ethical standards of the responsible committee on human experimentation (institutional and national) and with the Helsinki Declaration of 1964 and its later amendments. Informed consent was obtained from all patients for being included in the study.

### Animal studies

This study did not include any animal experiments.
